# Factors associated with physical activity policy and practice implementation in British Columbia’s childcare settings: a longitudinal study

**DOI:** 10.1186/s12889-023-16502-0

**Published:** 2023-08-29

**Authors:** Claire N. Tugault-Lafleur, Patti-Jean Naylor, Valerie Carson, Guy Faulkner, Erica Y. Lau, Luke Wolfenden, Louise C. Mâsse

**Affiliations:** 1 School of Nutrition Sciences, Faculty of Health Sciences, University of Ottawa, 25 University Private, Ottawa, ON K1N 6N5 Canada; 2 School of Exercise Science, Physical and Health Education, Faculty of Education, University of Victoria, Victoria, BC V8W 3P1 Canada; 3 Faculty of Kinesiology, Sport, and Recreation, University of Alberta, 8840-114 St., Edmonton, AB T6G 2H9 Canada; 4 School of Kinesiology, University of British Columbia, 210-6081 University Blvd, Vancouver, BC V6T 1Z1 Canada; 5 Department of Emergency Medicine, Faculty of Medicine, University of British Columbia, 828 West 10th Avenue, Vancouver, BC V5Z 1M9 Canada; 6 School of Medicine and Public Health, University of Newcastle, University Drive, Callaghan, NSW 2308 Australia; 7 School of Population and Public Health, Faculty of Medicine, University of British Columbia, Vancouver, BC V6T 1Z3 Canada; 8 BC Children’s Hospital Research Institute, Oak Street, Vancouver, BC F508-4490 Canada

**Keywords:** Physical activity, Natural policy experiment, Childcare, Canada

## Abstract

**Background:**

In 2016–17, the government of British Columbia (BC) enacted a mandatory policy outlining Active Play Standards (AP Standards) alongside a capacity building initiative (Appetite to Play) focused on implementing policies and practices to support physical activity in childcare centres. We aimed to identify factors at the provider and organizational levels as well as attributes of the Standards hypothesized to influence implementation (i.e., changes in policies and practices).

**Methods:**

We conducted surveys before (2016–2017) and after (2018–2019) enforcement of the AP Standards among 146 group childcare centres across BC. The 2018–19 surveys measured theoretically based constructs associated with implementation of policies and practices (9 childcare- and 8 provider- level characteristics as well as 4 attributes of the licensing standards). Characteristics that were associated in simple regression models were entered in multivariable regression models to identify factors associated with policy and practice changes related to fundamental movement skills (FMS), screen time, total amount of active play (AP) and total amount of outdoor AP from baseline to follow-up.

**Results:**

In multivariable analyses, higher staff capacity (OR = 2.1, 95% 1.2, 3.7) and perceived flexibility of the standards (OR: 3.3, 95% 1.5, 7.1) were associated with higher odds of a policy change related to FMS. Higher staff commitment to the AP standards was associated with a higher odds of policy changes related to screen time (OR = 1.6, 95% CI: 1.1, 2.4) and amount of AP (OR: 1.5, 95% 1.0, 2.3). Higher institutionalization of PA policies was associated with a higher odds of policy changes related to the amount of AP (OR: 5.4, 95% CI: 1.5, 20). Higher self-efficacy was associated with a higher odds of policy changes related to outdoor AP (OR = 2.9, 95% 1.1, 7.8). Appetite to Play training was a positively associated with practice changes related to FMS (β = 0.5, 95% CI: 0.1, 0.9).

**Conclusions:**

A hierarchy of theoretically defined factors influenced childcare providers’ implementation of the AP Standards in BC. Future research should test the feasibility of modifying these factors to improve the implementation of PA policy and practice interventions in this setting.

**Supplementary Information:**

The online version contains supplementary material available at 10.1186/s12889-023-16502-0.

## Background

Ensuring children get sufficient and quality physical activity (PA) is key to a healthy life [1, 2]. It is well established that children need regular, diverse, and vigorous PA for optimal development [3, 4]. Promoting sufficient and a variety of PA opportunities is important from a life course perspective [5] given evidence suggests that PA at a younger age is predictive of better PA and motor competence at an older age [6]. The current Canadian 24-Hour Movement Guidelines for the Early Years (0–4 year) states preschool children (3–4 years) should engage in a minimum of 180 min of any-intensity PA per day (of which at least 60 min should be energetic play) and screen time should be limited to no more than 60 min/day [7]. In the last nationally representative assessment that incorporated accelerometry data, 62% of 3- to 4-year-olds in Canada were meeting the PA recommendations within the Canadian 24-Hour Movement Guidelines for the Early Years [5]. However, only 24.4% of children met the screen time recommendation.

Childcare centers are key settings for health promotion and obesity prevention, as these services provide access to many children for prolonged periods of time. In Canada, more than half of children under age 5 attend childcare centers and of these children, 70% are in full-time (at least 30 h per week) childcare [8]. Therefore, institutional regulations related to PA and sedentary behaviour have the potential of reaching a large proportion of children. Indeed, determining the effectiveness of childcare-based policies for increasing children’s PA levels are part of the top ten research needs in changing children’s behaviours [9].

The impact of health promotion initiatives depends on the degree to which current PA polices and practices are implemented in practices. To realize their potential impact, health-promoting policies and practices must be implemented at scale [10]. However, implementation of best practice guidelines for PA in young children varies widely, despite best practice recommendations [11, 12].

In Canada, the childcare sector is not regulated by one universal regulatory body. Instead, regulations are set by individual provinces and territories [13]. Although all provinces have general recommendations to afford gross motor movement, most provinces give no specific requirements for how much and at what intensity, and few provinces and territories have specific standards related to sedentary behaviours [13, 14]. Moreover, despite having provincial regulations related to PA in childcare, a cross-sectional survey of Canadian childcare centers revealed that only 44% of centers have written center-level PA policies [12].

Canadian studies examining the effectiveness of policy interventions on changing childcare PA policies and practices are rare. However, in Alberta, Carson et al. compared changes in children’s PA levels before and after the release of new accreditation standards and compared these changes with those from children in Ontario (a control province which did not experience any PA policy changes over the same period) [15]. Overall, they found no differences in PA changes by province, suggesting that the accreditation standard criterion alone was insufficient to change PA behaviours. In the neighboring province of British Columbia (BC), our team previously reported on the level of implementation of new Active Play Standards (AP Standards) and the large difference in center-level PA, sedentary and healthy eating policies observed from 2016–17 to 2018–19 [16]. The AP Standards, introduced in 2016 by the provincial government of BC, targeted the following areas: 1) a minimum of 60 min per day of outdoor AP; 2) the incorporation of FMS and injury prevention in all AP activities; 3) limits on screen time and prolonged sitting; 4) role modeling by staff of AP and screen time; and 5) the development of written facility-level AP and screen time policies [17]. Unique to the BC policy change was also a large capacity-building initiative for early years providers, entitled Appetite to Play, that was initiated in the summer of 2017 and aimed to support the implementation of the AP Standards [18]. The purpose of Appetite to Play (https://appetitetoplay.com/) was to ensure early years providers had sufficient capacity to implement evidence-based policies, practices, and environments to support physical activity and healthy eating, and support compliance with the AP Standards [18]. Previous research has highlighted the widespread roll-out of the initiative which covered half of BC municipalities and was estimated to reach ~ 2700 early year providers over 18 months [18]. Finally, a noteworthy component of the BC policy implementation intervention was the use of licensing officers who were tasked to monitor the implementation of the AP Standards.

Current scale-up and implementation frameworks reflect the importance of strengthening organizational capacity and supporting implementation of polices and best practices [10, 19, 20]. Theoretical frameworks and reviews of empirical evidence from other settings suggest that a range of factors may facilitate the implementation of evidence-based programs by organisations. For example, Damschroder’s Consolidated Framework for Implementation Research (CFIR) is composed of constructs theorised to be associated with implementation across five major domains: intervention characteristics (e.g., cost and perceived complexity); outer setting factors (e.g., external policies and peer behavior); inner setting factors (e.g., alignment with organisational values and access to information and support); characteristics of the individuals (e.g., self-efficacy); and the process of implementation (e.g., planning) [19]. A 2020 systematic review and meta-analysis examining the effectiveness of strategies to improve the implementation of PA and healthy eating policies, practices, and programs by childcare services suggest that implementation strategies such as continuous quality improvement, educational materials, performance monitoring can lead to improvements in implementation outcomes [21]. Yet developing strategies to effectively improve childcare service adherence to PA standards requires understanding which factors may impede or promote implementation.

The release of new PA standards (AP Standards) in BC provided a unique opportunity to identify factors that supported implementation (i.e., changes in PA and sedentary behaviour policies and practices within childcare centers). In this paper, we use data from licensed group childcare centers that participated in both data collection periods (i.e., pre and post implementation of the new AP Standards) to explore factors (e.g., PA capacity, organizational climate, behavioural capability of the care providers) associated with implementation. We chose to examine factors associated with changes in four types of policies and practices specifically targeted by the AP Standards: FMS, amount of daily AP, limits on screen time, and amount of outdoor AP.

## Methods

### Design and participants

This study used data from a longitudinal cohort embedded within a larger repeat cross-sectional study, which surveyed managers and staff from licensed group childcare facilities before and after the enforcement of the AP Standards [22]. The first wave of survey recruitment started from October 2016 and ended in August 2017. At baseline, only the size of the play space and providing opportunities for gross motor movements were specified in the provincial regulations [23]. The AP Standards are mandatory and were officially released in July 2016 but were not enforced (i.e., monitored through inspections) until a year later, in the summer of 2017. The second wave of survey recruitment took place from October 2018 to September 2019, approximately one year after the enforcement of the AP Standards and the launch of Appetite to Play.

Initially, licensing officers reached out to early years group childcare managers (managing 30 months to 5-year-old children) via email. The research team followed up with potential participants through direct mail, email, and phone calls. We also compiled a list of licensed childcare centers using publicly available facility contact information. To further broaden our recruitment efforts, we distributed invitation letters through childcare resource and referral agencies, and early childhood educator newsletters. Childcare managers were asked to forward the staff invitation and survey links to their staff. Childcare center managers who did not respond to initial and follow-up email invitations were sent a paper copy of both the manager and two staff surveys with pre-paid postage-stamped envelopes for return. Managers who had not yet responded also received phone calls from our research team and were offered the choice of web-based or paper survey for themselves and their staff. Respondents were eligible to participate if their facility cared for children aged 2 ½ -5 years old, was licensed for group childcare, preschool (offering full days), and/or multi-age childcare, and if the respondent was a manager or a staff overseeing or caring for children aged 2 ½—5 years old.

All methods were performed in accordance with the relevant guidelines and regulations and the study was approved by the University of Victoria and the University of British Columbia (UBC) Harmonized Research Ethics Review Board (BC16-128 and H18-01434). Respondents gave their implicit and informed consent by answering the Early Years survey.

### Measures

At both time points, managers and staff completed an online survey which included questions about their PA and sedentary policies and practices as well as demographics. Questions about the PA/sedentary environment were adapted from the validated Environment and Policy Evaluation and Observation Self-Report (EPAO-SR) instrument [16] but shortened to enhance response rate and reflect the broader surveillance-oriented survey approach of our study. We developed additional survey items about daily practices related to the new AP Standards and BC-specific childcare characteristics based on feedback from a provincial early year’s healthy eating and PA resource advisory committee, the research team, and a pilot group of early childhood educators (*n* = 7). In 2018–19, some survey items were also refined to ensure that the questions measured the policies and practices targeted by the AP Standards and addressed implementation factors at the center- and provider- level which were hypothesized to play a role in implementation. The factorial structure of the constructs were tested with confirmatory factor analysis (CFA), drawing from a larger sample of childcare providers within BC. Overall fit of the CFA were analyzed using the following criteria as there are no agreed standards: Steiger’s Root Mean Square Error of Approximation, with an upper value of 0.08 to 0.10 indicative of a good fit; the Comparative Fit Index with values ≥ 0.95 suggestive of an acceptable fit; and the Standardized Root Mean Square Residual with a value <  = 0.05 suggestive of a good fit [24].

### Facility-level policies

At both time points, respondents were asked whether their center had a written PA/sedentary policy related to: 1) offering opportunities for FMS (i.e., either locomotor skills (e.g., running, hopping, jumping), balance and/or manipulative skills (throwing, catching, kicking)); 2) the amount of time children could play with screens (watch television/video each day, computer, games; 3) the total amount of AP daily; and 4) the total amount of outdoor AP daily. Respondents could select responses ranging from “No policy”, “Yes, no written policy but general practice”, “Yes, written policy”, or “N/A”. Responses were dichotomized into “Yes” (written policy present) or “no written policy” (grouping the “Yes, not written policy, but general practice” and the “No written policy”) together.

When more than one manager or more than one staff from the same facility responded to the survey (69% of the centers), their answers were aggregated across duplicate responses by role (i.e., across all managers or across all staff). If agreement among respondents of a given role (managers or staff) was greater than 66%, their policy answer was rounded to either no written policy (= '0') or written policy (= '1'). If agreement among respondents was below 66%, their survey response was coded as missing. If both manager(s) and staff within a facility provided policy responses, we prioritized the manager(s)’ response(s). However, if no manager within a center provided policy responses, we supplemented our policy data with staff response(s) when available. Change scores were then computed, classifying centers as either no change (no written policy at baseline or follow-up) or a positive change (no written policy at baseline but a written policy at follow-up). Those centers who already had a written policy at baseline were excluded from the analyses as no change score could be computed.

### Facility-level practices

At both time points, respondents were asked how frequently children in their program: 1) engaged in activities to encourage fundamental movement skills; 2) spent 30 min or less on screens per day; 3) engaged in at least 120 min of active play and PA per day (60 min for 1/2 day); 4) participated in at least 60 min of outdoor AP per day. Response option ranged from “rarely/never”, “infrequently”, “some days”, “most days (3/4 days/week)”, to “Daily” and were coded using a 1–5 Likert scale where the higher the score, the greater the frequency of a given practice.

When more than one manager or more than one staff from the same facility responded to the survey, the practice scores were averaged across all respondents by role (i.e., across all managers or across all staff). While managers may believe their frontline staff behave in a certain way, the nature of their administrative role and limited direct experience with the classroom may prevent them from providing a reliable account of what is occurring in the center [12, 25, 26]. If both manager(s) and staff within a facility provided practice responses, we therefore prioritized the staff’s response(s). When no staff answered the survey, we supplemented the reported practice data with manager response(s) when available. Change/differences in mean scores for practices were calculated for each center by subtracting scores from baseline from scores at follow-up.

### Characteristics of early years group childcare providers

The Early Years follow-up survey (2018–19) was enhanced to comprehensively assess a set of theoretically based constructs associated with implementation of policies and practices among early year group childcare centers. Drawing from Damschroder’s CFIR (Damschroder et al., 2009), the following 9 constructs were assessed: organizational climate (4 items; Cronbach α = 0.72), PA culture (4 items; Cronbach α = 0.82), PA capacity (space and equipment) (3 items; Cronbach α = 0.67), PA capacity (material) (1 item), PA capacity (staff) (2 items; Cronbach α = 0.68), PA capacity (time) (2 items; Cronbach α = 0.68), the overall commitment from staff (at the center-level) to the AP Standards (1 item), implementation support (7 items; Cronbach α = 0.84) and level of institutionalization (3 items; Cronbach α = 0.75). For each question, the questionnaire provided a 1–5 Likert response scale ranging from “strongly disagree” to “strongly agree”, except for the 4 organizational climate items (collegial vs. hostile, supportive vs. unsupportive, accepting of change vs. cautious to change, friendly vs. unfriendly) which used a semantic differentials scale (+ 3, + 2, + 1, 0, -1, -2, -3) where a positive score indicates a more desirable organisational climate. Additional File 1 shows adequate fit was achieved for organizational climate and PA culture (2-factor solution), PA capacity (3-factor solution) and implementation support and level of institutionalization (2-factor solution).

At the provider level, a total of 8 constructs were assessed: knowledge of the AP Standards, awareness of the Appetite to Play intervention, whether the provider had completed the Appetite to Play training, whether the provider had previously completed any PA or physical literacy training (other than Appetite to Play), whether the staff at the facility felt motivated to implement the AP Standards, and whether the provider felt they had the skills to facilitate playful physical activities that build FMS. All these constructs were assessed using a “yes/no” response option. Participants were also asked about their use of the Appetite to Play resources (2 items, “Are you using the Appetite to Play Community Board or social media page?”, “Are you currently using the Appetite to Play resources such as the planning tools, physical activity games, tips or ideas?”), with a 1–4 Likert response scale ranging from “never”, “at least once a month”, “at least once a week”, and “multiple times per week”. Finally, the provider’s self-efficacy toward PA (4 items, e.g. “I am capable of planning daily fundamental movement skills according to the Active Play standards”) were assessed using a 1–5 Likert response scale ranging from “strongly disagree” to “strongly agree”. Responses were coded so that a higher score indicated higher frequency of use of the Appetite to Play resources or higher levels of self-efficacy toward PA, respectively. Internal reliabilities for these 2 scales were deemed acceptable (Cronbach’s α scores = 0.76 and 0.72 for use of Appetite to Play resources and self-efficacy toward PA, respectively). A 1-factor model for self-efficacy towards PA showed adequate fit (see Additional file 1).

Four attributes of the AP Standards were assessed: flexibility & triability of the AP Standards (3 items, e.g. “There are many ways I can implement and meet the Active Play standards”), their outcome expectations (3 items, e.g., “Implementation of the Active Play Standards and activities can increase children’s interests in physical activity”), their relative advantage (2 items, e.g. “I believe that the Active Play standards plus Appetite to Play resources provided are easier to use than other physical activity or skill programs”) and finally, the acceptability of the AP Standards (2 items, e.g. “I find the Appetite to Play resources appealing”). For each question, the questionnaire provided a 1–5 Likert response scale ranging from “strongly disagree” to “strongly agree”. Scores were coded so that a higher score indicated higher endorsement for each construct. Internal reliabilities for these scales were also deemed acceptable or good, with Cronbach’s α scores for all constructs ranging from 0.76 to 0.85. A model with a 4-factor solution resulted in adequate fit (see Additional File 1).

For constructs assessed with at least 2 or more items (e.g., organizational climate, PA culture, level of implementation support, flexibility & triability of the AP Standards, self-efficacy toward PA), an average score was computed across items. If a center had more than one respondent complete the survey, an average score for the construct was computed for the center across respondents. Descriptive statistics for each construct among centers surveyed at time 2 are shown in Table 1.

**Table 1:** Descriptive statistics of the center-, provider- level characteristics and attributes of the Active Play Initiative hypothesized to be influence changes in center-level physical activity (PA) and sedentary behaviour policies and practices (measured in the 2018–19 follow-up surveys) (n = 146 centers)

	**n**^**a**^	**Range**	**Mean (SD) / %**
**Center-level characteristics**			
Organizational climate (4 items)	139	3.1–7	6.3 (0.8)
PA culture (4 items)	146	2.5–5	4.4 (0.5)
PA capacity (space and equipment) (4 items)	146	2–5	4.3 (0.6)
PA capacity (materials) (1 item)	146	1–5	4.0 (0.8)
PA capacity (staff) (2 items)		1.8–5	4.0 (0.8)
PA capacity (time) (2 items)	146	2–5	4.5 (0.7)
Commitment from staff to the AP Standards (1 item)	146	1–5	4.0 (1.1)
Implementation support (7 items)	146	1.6–5	3.4 (0.7)
Level of institutionalization (3 items)	137	0–1	0.4 (0.4)
**Provider-level characteristics**			
Aware of the AP Standards, % yes	140	-	98.0
Aware of the Appetite to Play initiative, % yes	136	-	81.6
Attended Appetite to Play training, % yes	145	-	25.5
Completed any physical literacy training (excluding Appetite to Play) training, % yes	145	-	36.6
Staff motivation to implement AP Standards, % yes	144	-	93.1
Behavioural capability (1 item), % yes	144	-	94.4
Use of Appetite to Play resources (2 items)	145	0–3	0.6 (0.7)
Self-efficacy around PA and physical literacy (4 items)	143	2.7–5	4.5 (0.5)
**Attributes of the AP Standards**			
Flexibility/trialability (3 items)	142	2.3–5	4.1 (0.6)
Outcome expectations (3 items)	142	3–5	4.6 (0.4)
Relative advantage (2 items)	142	2–5	3.8 (0.7)
Acceptability (2 items)	142	1–5	3.7 (0.7)

*PA* Physical activity. *SD* Standard deviation.

^a^Number of centers with non-missing data for each construct

### Other area-level characteristics

Area-level sociodemographic information were collected to be included as covariates all regression models. Population size, percentage of the population with some post-secondary education and median household income were obtained from the Statistics Canada 2016 Census and linked to childcare site data using postal codes.

### Statistical analyses

Simple descriptive statistics (means and standard deviations (SD), proportions or frequencies (%)) were used to describe center- and provider- level characteristics, prevalence of centers with written policies and frequency of practices. Simple logistic and linear regression models were fitted to investigate associations between each of the theoretically derived factors measured at follow-up (i.e., center- and provider-level characteristics, specific attributes of the AP Standards) (independent variables) and changes in policies and frequency of reported practices between baseline and follow-up (dependent variables). All models were adjusted for area-level sociodemographic characteristics (population count, proportion of the population with some post-secondary education and total household income).

Characteristics that showed evidence of an association in simple regression models were entered in a multivariable model to identify the most salient factors associated policy and practice changes over time. A screening criterion of *p* < 0.05 was adopted to determine which variables would be included in the multivariable model. Since this study aimed to examine which characteristics at the organizational- and provider- level were associated with changes in policies and practices, it was important to evaluate multicollinearity within a model. Multicollinearity was assessed by examining the variance inflation factor (VIF) (where any values > 2.5 was considered indicative of multicollinearity) [27] and bivariate correlations (where multicollinearity was investigated for correlations > 0.50) [28]. The impact of multicollinearity between predictors in multivariable models were assessed as follows. First, all variables that were significant (*p* < 0.05) were included. Second, we assessed whether the removal of any collinear variable(s) changed the result (meaning the significance of the variable changed and became significant). If this was the case, the collinear variable was removed from the final model (this is denoted by a footnote in the Tables).

Assumptions for the multivariable linear regression models (linearity, heteroscedasticity, normality of residuals) were assessed through bivariate scatterplots, the Breusch-Pagen test, and Kernal density and standardised normal probability plots, respectively. To handle the missing data on policies and practices, multivariate multiple imputation was used. Data augmentation, a Bayesian iterative Markov Chain Monte Carlo procedure [29], was used to approximate the distribution of the missing data creating 30 imputed datasets. Mean odds ratios (for logistic regression models) and regression coefficients (unstandardized betas) were computed from the 30 imputed datasets. Statistical significance was set at *p* < 0.05. All analyses were run using Stata version 15 [30].

## Results

A total of 246 and 228 providers from 146 centers answered the surveys at baseline and follow-up, respectively. The sociodemographic profile of respondents was overall similar from baseline to follow-up, with slightly more managers compared to staff responding to the surveys (Table 2). As previously reported [22], there was a substantial change in the proportion of centers with PA conducive policies.

**Table 2:** Demographic and center-level characteristics before and after the enforcement of an AP Standards among early year group childcare providers in British Columbia, Canada

	**Survey year**			
	**2016–17**		**2018–19**	
**Respondents’ characteristics**				
Respondents, n (%)	246 (51.9)		228 (48.1)	
Sex, n (%)				
Female	237 (96.3)		202 (91.8)	
Male	5 (2.0)		6 (2.7)	
Prefer not to disclose	4 (1.6)		12 (5.4)	
Age group, n (%)				
< 30 years	42 (16.6)		31 (13.6)	
30–39 years	63 (25.6)		77 (33.8)	
40–49 years	69 (28.1)		51 (22.7)	
> 50 years	73 (29.6)		69 (30.3)	
Role, n (%)				
Manager	141 (57.3)		131 (57.3)	
Staff	105 (42.7)		97 (42.5)	
Years working at center, n (%)				
1–4 years	62 (25.2)		62 (27.2)	
≥ 5 years	184 (74.8)		166 (72.8)	
**Center-level characteristics (*n* = 146 centers in both survey cycles)**				
**Policies, n (%)**				
Policy on fundamental movement skills				
No written policy	112 (87.5)		59 (43.4)	
Written policy	16 (12.5)		77 (56.6)	
Policy on screen time				
No written policy	88 (69.4)		31 (22.6)	
Written policy	38 (30.2)		106 (77.4)	
Policy on total amount of AP				
No written policy	79 (63.2)		39 (28.7)	
Written policy	46 (36.8)		97 (71.3)	
Policy on outdoor AP				
No written policy	68 (54.0)		29 (21.0)	
Written policy	58 (46.0)		109 (79.0)	
**Reported daily practices**^**a**^	**n [range]**	**mean (SD)**	**n [range]**	**mean (SD)**
Opportunities for fundamental movement skills	127 [1, 5]	4.7 (0.7)	131 [1, 5]	4.7 (0.7)
Less than 30 min of screen time	125 [1, 5]	3.0 (1.9)	129 [1, 5]	3.9 (1.7)
Minimum 120 min of PA and AP	126 [1, 5]	4.6 (0.8)	131 [1, 5]	4.7 (0.6)
Min 60 min of outdoor AP	121 [1, 5]	4.7 (0.7)	130 [2, 5]	4.9 (0.4)

*AP* Active Play. *PA* Physical activity. *SD* Standard deviation.

^a^All practice items were measured using a 1–5 Likert scale ranging from “Rarely/Never” (1), “infrequently” (2), “some days (1–2/week)” (3), most days (3–4/week) (4), to “Daily” (5). The higher the score, the more frequent the behaviour

Table 3 displays the strength of associations between 9 center- and 8 provider-level characteristics as well as 4 attributes of the Standards with odds of developing written policies from baseline to follow-up. In simple regression models, all center-level factors (organisational climate, PA culture, PA capacity, commitment at the center-level, implementation support, and institutionalization of policies), 1 provider-level factor (self-efficacy) and 3 out of 4 attributes of the Standards (perceived flexibility, outcome expectations and relative advantage) were each independently associated with developing written PA/sedentary policies from baseline to follow-up for at least one of the 4 types of policies examined (*P* < 0.05 for all factors).

**Table 3:** Factors associated with odds of developing written policies related to FMS, screen time, total amount of AP and outdoor AP in a cohort of childcare centers (n = 146): simple and multivariable logistic regression models

	**Policy on FMS**		**Policy limiting screen time**		**Policy on total daily amount of AP**		**Policy on amount of outdoor AP**	
	**Simple regression models**	**Multivariable model**	**Simple regression models**	**Multivariable model**	**Simple regression models**	**Multivariable model**	**Simple regression models**	**Multivariable model**
	**Odds Ratios (OR) (95% Confidence Intervals (CI)), p-value**							
**Center characteristics**								
Organizational climate	1.4 (1.0, 2.4), *p* = 0.054	1.2 (0.7, 1.9), *p* = 0.539	**1.7 (1.0, 2.7), *p* = 0.039**	1.1 (0.7, 2.2), *p* = 0.431	**1.8 (1.1, 3.0), *p* = 0.013**	1.4 (0.8, 2.4), *p* = 0.287	**2.5 (1.5, 4.2), *p* < 0.001**	1.8 (1.1, 3.2), *p* = 0.070
PA culture	**3.0 (1.5, 5.9), *p* = 0.002**	-a	2.1 (1.0, 4.5), *p* = 0.054		1.9 (1.0, 3.9), *p* = 0.066		2.1 (0.9, 4.2), *p* = 0.075	
Capacity (space and equipment)	**1.7 (1.5, 4.8), *p* = 0.001**	-a	1.1 (0.6, 2.1), *p* = 0.663		1.6 (0.9, 2.7), *p* = 0.127		**2.4 (1.3, 4.5), *p* = 0.005**	1.8 (1.0, 2.8), *p* = 0.486
Capacity (materials)	1.8 (1.2, 3.0), *p* = 0.007	-a	1.1 (0.7, 1.8), *p* = 0.642		**1.7 (1.1, 2.6), *p* = 0.029**	0.9 (0.5, 1.7), *p* = 0.787	1.6 (1.0, 2.6), *p* = 0.076	
Capacity (staff)	**2.4 (1.8, 5.8), *p* = < 0.001**	**2.1 (1.2, 4.0), *p* = 0.024**	**1.9 (1.0, 3.4), *p* = 0.042**	1.4 (0.7, 2.8), *p* = 0.278	**2.1 (1.2, 3.7), *p* = 0.008**	-b	**2.4 (1.3, 4.6), *p* = 0.006**	-c
Capacity (time)	**2.1 (1.2, 3.6), *p* = 0.013**	-a	1.5 (0.8, 2.8), *p* = 0.159		1.5 (0.8, 2.6), *p* = 0.179		**2.6 (1.4, 5.0), *p *= 0.003**	-c
Staff commitment to AP Standards	1.1 (0.8, 1.4), *p* = 0.687		**1.7 (1.2, 2.5), *p* = 0.002**	**1.6 (1.1, 2.4), *p* = 0.012**	**1.5 (1.0, 2.0), *p* = 0.027**	**1.5 (1.0, 2.3), *p* = 0.046**	**1.5 (1.1, 2.1), *p* = 0.025**	1.2 (0.8, 1.9), *p* = 0.399
Implementation support	**3.7 (2.0, 6.9), *p* < 0.001**	-a	1.1 (0.7, 2.1), *p* = 0.591		**2.4 (1.3, 4.3), *p* = 0.003**	-b	1.8 (1.0, 3.3), p = 0.055	
Level of institutionalization	1.8 (0.7, 4.6), *p* = 0.250		1.0 (0.3, 3.0), *p* = 0.982		**4.7 (1.4, 15.3), *p* = 0.012**	**5.4 (1.5, 20.0), *p* = 0.011**	2.6 (0.8, 8.9), *p* = 0.132	
**Provider characteristics**								
Knowledge of AP Standards^1^	2.8 (0.2, 32.9), *p* = 0.450		2.1 (0.2, 25.5), *p* = 0.565		N/A		N/A	
Knowledge of Appetite to Play initiative^1^	0.9 (0.4, 2.2), *p* = 0.840		1.7 (0.6, 4.5), *p* = 0.316		2.0 (0.8, 5.0), *p* = 0.138		0.9 (0.3, 2.8), *p* = 0.925	
Staff motivation to implement AP Standards	1.5 (0.4, 5.6), *p* = 0.518		2.7 (0.7, 10.1), *p* = 0.153		1.2 (0.3, 5.0), *p *= 797		1.0 (0.2, 4.8), *p* = 0.960	
Behavioural capability	0.8 (0.2, 3.7), *p* = 0.826		1.3 (0.2, 6.8), *p* = 0.775		1.6 (0.4, 7.2), *p* = 0.538		1.2 (0.2, 6.6), *p* = 0.809	
Self-efficacy toward PA	**4.0 (1.9, 8.4), *p* < 0.001**	-a	**2.2 (1.0, 4.9), *p* = 0.055**		**2.7 (1.3, 5.7), *p* = 0.011**	1.5 (0.5, 4.3), *p* = 0.414	**4.8 (2.0, 11.4), *p* < 0.001**	**2.9 (1.1, 7.8), *p* = 0.031**
Attended Appetite to Play training^1^	1.6 (0.7, 3.7), *p* = 0.219		2.8 (0.9, 8.7), *p* = 0.077		1.1 (0.5, 2.6), *p* = 0.835		2.0 (0.6, 5.8), *p* = 0.215	
Physical literacy training^1^	2.0 (1.0, 4.2), *p* = 0.056		1.3 (0.6, 3.0), *p* = 0.538		1.7 (0.7, 3.8), *p* = 0.208		1.3 (0.5, 3.3), *p* = 0.525	
Use of Appetite to Play resources^1^	1.6 (1.0, 2.7), *p* = 0.052		1.5 (0.8, 2.8), *p* = 0.175		1.7 (1.0, 3.2), *p *= 0.072		1.1 (0.6, 2.1), *p* = 0.644	
**Attributes of the AP Standards**								
Flexibility/trialability	**4.7 (2.3, 9.6), *p* < 0.001**	**3.3 (1.5, 7.1), *p* = 0.002**	1.2 (0.6, 2.5), *p* = 0.529		**2.2 (1.2, 4.3), *p* = 0.017**	-b	1.9 (1.0, 3.9), *p* = 0.069	
Outcome expectations	**3.1 (1.4, 7.0), *p* = 0.006**	-a	1.8 (0.7, 4.3), *p* = 0.232		2.2 (0.9, 5.2), *p* = 0.069		**3.2 (1.2, 8.4), *p *= 0.016**	-c
Relative advantage	**2.4 (1.4, 4.1), *p* = 0.001**	-a	1.1 (0.6, 1.9), *p* = 0.805		**1.8 (1.1, 3.2), *p* = 0.030**	1.6 (0.8, 3.1), *p* = 0.173	1.5 (0.8, 2.8), *p* = 0.169	
Acceptability	1.4 (0.9, 2.2), *p* = 0.171		1.0 (0.6, 1.7), *p* = 0.890		1.1 (0.6, 1.8), *p* = 0.810		1.0 (0.6, 1.8), *p* = 0.953	

*AP* Active Play. *FMS* Fundamental movement skills. *PA* Physical Activity. Covariates included area-level community variables (population size, median income, and percent of individuals with some post-secondary education)

^1^ Dichotomous characteristic (yes/no, with ‘no’ as the reference group)

^a^ PA culture, capacity (time, materials, space and equipment), implementation support, self-efficacy around PA, outcome expectation and relative advantage were removed from the final model due to multicollinearity among other factors predicting odds of having written policies on fundamental movement skills

^b^ PA capacity (staff), implementation support and flexibility/triability were removed from the final model due to multicollinearity among other factors within the model predicting odds of having written policies on total amount of active play

^c^ Capacity (staff and time) and outcome expectations were removed from the final model due to multicollinearity among other factors within the model predicting odds of having written policies on outdoor active play

Results from multivariable regression analyses for each type of policy is also shown in Table 3, after removing some predictors due to multicollinearity (see footnotes in Table 3). Greater staff capacity (OR = 2.1, 95% 1.2, 3.7) and perceived flexibility of the Standards at follow-up (OR: 3.3, 95% 1.5, 7.1) were associated with a higher odds of policy changes related to FMS but only after removing collinear factors (PA culture, PA capacity related to space and equipment, materials, time, implementation support and self-efficacy, outcome expectation and relative advantage). In multivariable analyses, higher staff commitment to the AP Standards at follow-up was associated with a higher odds of a positive policy change related to limiting screen time (OR = 1.6, 95% CI: 1.1, 2.4). Both higher staff commitment to the AP Standards (OR: 1.5, 95% 1.0, 2.3) and higher institutionalization of PA policies at follow-up (OR: 5.4, 95% CI: 1.5, 20) were associated with higher odds of developing written policies related to the amount of AP, but only after removing other collinear predictors (PA capacity related to staff, implementation support, and flexibility/triability of the Standards). Finally, higher self-efficacy toward PA at follow-up (OR = 2.9, 95% 1.1, 7.8) was associated with higher odds of a policy change related to outdoor AP, but only after removing other collinear predictors (PA capacity related to staff, time and outcome expectations).

Table 4 displays the strength of associations between center- and provider-level characteristics as well as attributes of the Standards and practice changes in simple and multivariable regression models. Compared to policy changes, fewer factors were found to be predictive of practice changes. In simple regression models, 5 out of the 10 center-level factors (organizational climate, PA capacity (materials, time), staff commitment and implementation support) and 2 provider-level factors (self-efficacy and previous Appetite to Play training) were each independently associated with positive changes with at least one of the 4 PA and sedentary behaviour practices examined (*P* < 0.05 for all outcomes). However, greater capacity for space and equipment at follow-up was associated with a decrease (or a negative change) in practices related to the amount of AP and outdoor AP (*P* < 0.05 for both outcomes). In multivariable analyses, Appetite to Play training at follow-up was associated with an increase in practices related to FMS (β = 0.5, 95% CI: 0.1, 0.9) but only after removing collinear predictors (PA capacity related to time and implementation support).

**Table 4:** Factors associated with practice changes related to FMS, limiting screen time, total amount of AP and outdoor AP in a cohort of childcare centers (n = 146): simple and multivariable linear regression models

	**Fundamental movement skills**		**Limiting screen time**	** ≥ 120 min of active play**	** ≥ 60 min of outdoor active play**
	**Simple regression models**	**Multivariable regression model**	**Simple regression models**	**Simple regression models**	**Simple regression models**
**β coefficient (95% CI), p-value**					
**Center characteristics**					
Written PA policy	0.2 (-0.3, 0.6), *p* = 0.504		0.8 (-0.1, 1.7), *p* = 0.077	-0.2 (-0.5, 0.1), *p* = 0.245	-0.3 (-0.6, 0.1), *p* = 0.126
Organizational climate	**0.3 (0.1, 0.6), *p* = 0.011**	0.2 (-0.0, 0.5), *p* = 0.063	0.2 (-0.4, 0.7), *p* = 0.493	-0.0 (-0.2, 0.2), *p *= 0.811	0.1 (-0.1, 0.3), *p *= 0.575
PA culture	0.3 (-0.0, 0.7), *p* = 0.069		-0.7 (-1.5, 0.1), *p* = 0.086	0.0 (-0.3, 0.4), *p* = 0.815	-0.2 (-0.5, 0.1), *p* = 0.201
Capacity (space and equipment)	0.0 (-0.3, 0.4), *p* = 0.732		-0.6 (-1.2, 0.1), *p* = 0.096	**-0.3 (-0.6, -0.1), *p* = 0.019**	**-0.3 (-0.6, -0.1), *p* = 0.006**
Capacity (materials)	**0.3 (0.1, 0.6), *p* = 0.016**	0.2 (-0.1, 0.5), *p* = 0.116	0.4 (-0.8, 1.1), *p* = 0.164	-0.1 (-0.3, 0.2), *p *= 0.638	-0.1 (-0.3, 0.1), *p* = 0.440
Capacity (staff)	0.3 (-0.0, 0.6), *p* = 0.066		-0.2 (-0.8, 0.5), *p* = 0.618	-0.1 (-0.4, 0.1), *p* = 0.340	-0.2 (-0.4, 0.1), *p* = 0.227
Capacity (time)	**0.4 (0.1, 0.8), *p* = 0.014**	-a	-0.5 (-1.3, 0.2), *p* = 0.200	-0.1 (-0.4, 0.3), *p* = 0.601	-0.1 (-0.4, 0.2), *p* = 0.474
Staff commitment to AP Standards	0.1 (-0.1, 0.3), *p* = 0.187		**0.4 (0.1, 0.8), *p* = 0.016**	0.1 (-0.1, 0.2), *p* = 0.519	0.1 (-0.1, 0.2), *p* = 0.439
Implementation support	**0.3 (0.0, 0.6), *p* = 0.040**	-a	0.3 (-0.9, 0.3), *p* = 0.332	-0.1 (-0.3, 0.2), *p* = 0.615	-0.1 (-0.3, 0.1), *p* = 0.356
Level of institutionalization	0.1 (-0.5, 0.7), *p* = 0.707		-0.9 (-1.9, 0.3), *p* = 0.144	0.0 (-0.5, 0.5), *p* = 0.979	-0.1 (-0.5, 0.3), *p* = 0.507
**Provider characteristics**					
Knowledge of AP Standards^1^	-1.3 (-2.6, 0.0), *p* = 0.059		-0.8 (-3.8, 2.2), *p* = 0.614	0.2 (-1.1, 1.4), *p* = 0.770	0.1 (-0.9, 1.3), *p* = 0.749
Knowledge of Appetite to Play initiative^1^	0.3 (-0.2, 0.8), *p* = 0.221		-0.0 (-1.0, 1.0), *p* = 0.969	0.3 (-0.1, 0.8), *p* = 0.163	0.1 (-0.3, 0.4), *p* = 0.716
Staff motivation to implement AP Standards	0.6 (-0.1, 1.3), *p* = 0.089		0.6 (-0.9, 2.0), *p* = 0.344	0.2 (-0.4, 0.8), *p* = 0.471	0.0 (-0.5, 0.6), *p* = 0.882
Behavioural capability	0.6 (-0.4, 1.6), *p* = 0.255		0.3 (-1.5, 2.0), *p* = 0.779	0.1 (-0.7, 0.9), *p* = 0.810	-0.1 (-0.8, 0.7), *p* = 0.783
Self-efficacy toward PA	**0.6 (0.2, 1.0), *p* = 0.005**	0.2 (-0.2, 0.5), *p* = 0.116	-0.4 (-1.2, 0.4), *p* = 0.290	-0.0 (-0.4, 0.3), *p* = 0.847	0.0 (-0.3, 0.3), *p* = 0.828
Attended Appetite to Play training^1^	**0.6 (0.2, 1.0), *p* = 0.007**	**0.5 (0.1, 0.9), *p *= 0.019**	-0.1 (-1.0, 0.8), *p *= 0.848	0.1 (-0.3, 0.5), *p* = 0.715	-0.1 (-0.4, 0.3), *p* = 0.651
Physical literacy training^1^	0.1 (-0.3, 0.5), *p* = 0.686		0.1 (-0.7, 1.0), *p* = 0.737	0.1 (-0.3, 0.5), *p* = 0.595	-0.2 (-0.5, 0.2), *p* = 0.351
Use of Appetite to Play resources^1^	0.1 (-0.2, 0.4), *p* = 0.403		-0.3 (-1.0, 0.3), *p* = 0.294	0.1 (-0.2, 0.3), *p* = 0.581	-0.1 (-0.3, 0.1), *p* = 0.265
**Attributes of the AP Standards**					
Flexibility/trialability	0.1 (-0.1, 0.5), *p* = 0.248		-0.4 (-1.1, 0.2), *p* = 0.216	-0.1 (-0.4, -0.2), *p* = 0.358	0.1 (-0.5, 0.3), *p* = 0.527
Outcome expectations	0.5 (-0.1, 0.9), *p* = 0.080		-0.6 (-1.5, 0.3), *p* = 0.205	-0.0 (-0.4, 0.4), *p* = 0.858	0.1 (-0.3, 0.1), *p* = 0.184
Relative advantage	0.2 (-0.1, 0.5), *p* = 0.166		-0.3 (-0.9, 0.2), *p* = 0.234	-0.0 (-0.2, 0.3), *p* = 0.699	-0.1 (-0.3, 0.1), *p *= 0.380
Acceptability	0.2 (-0.0, 0.5), *p* = 0.063		-0.4 (-0.9, 0.1), *p* = 0.158	0.1 (-0.1, 0.3), *p* = 0.620	0.2 (-0.9, 1.3), *p* = 0.749

*AP* Active Play. *FMS* Fundamental movement skills. *PA* Physical Activity. No multivariable regression models were run for screen time, daily amounts of active play and outdoor active play since fewer predictors were associated with practice changes over time. Covariates included area-level community variables (population size, median income, and percent of individuals with some post-secondary education)

^1^ Dichotomous characteristic (yes/no, with “no” as the reference group)

^a^ Implementation support was removed due to its collinearity with PA capacity (materials). PA capacity (time) was removed due to its collinearity with self-efficacy

## Discussion

This study examined a wide range of theoretically defined factors hypothesized to influence the implementation of new AP Standards within childcare centers in BC, Canada. We found that factors within the inner (childcare) setting and characteristics of the individuals involved (childcare providers) were the most salient predictors of policy and practices changes while attributes of the intervention itself were not significant in multivariable models. While fewer factors were associated with practices changes, reporting previous Appetite to Play training emerged as a salient predictor of practice changes related to FMS.

Studies examining naturally occurring provincial policy change related to physical activity in childcare are rare, but our findings can be compared with those of a similar study in the neighboring province of Alberta, where the government introduced a PA accreditation standard in 2013 (which was phased in until 2019) [15]. In contrast to the policy intervention in Alberta, the new AP Standards had a specific benchmark for physical activity (i.e., 60 min per day) and extensive training was provided through the Appetite to Play initiative to help improve implementing of the new Standards, along with enforcement from BC licensing officers. These findings point to the importance of the quality of the training and resources to support directors and educators along with policy enforcement when implementing similar policy changes targeting the childcare PA environment.

In simple regression models, we found that all nine center-level characteristics were individually associated with greater odds of having a written PA/sedentary policy related to either FMS, screen time, total amount of AP or outdoor AP. Some factors within the inner setting predicted greater odds of several types of policies. For example, a more positive (i.e., more collegial, supportive, accepting of change and friendly) organizational climate predicted the odds of having written policies related to screen time, amount of AP and outdoor AP while greater PA capacity among staff was associated with greater odds of having all four types of policies. Greater implementation support was associated with greater odds of having center-level policies related to FMS and daily AP. These findings highlight the importance of targeting characteristics within the inner setting (PA capacity related to staff, infrastructure, schedules, organizational support, and climate) to facilitate the implementation of the AP Standards within childcare centres in BC.

In multivariable models predicting odds of creating a written policy at follow-up, many constructs were dropped due to collinearity between predictors, which highlights the complexity of studying these determinants. Many factors were significantly associated with policy/practice changes in the simple regression models, but these became non-significant in the adjusted models. For example, some provider-level characteristics such as self-efficacy were collinear with characteristics of the childcare setting such as PA culture. However, one might note that being non-significant did not mean they were unimportant. They might function as preceding factors/catalysts in promoting change through another factor [31], speaking to the importance of future studies to identify underlying mechanisms.

These results highlight the significance of factors within the social environment to improve policy implementation. In our more parsimonious model predicting policy changes, only factors related to the social environment were retained: organizational climate, the overall level of staff commitment, and PA staff capacity. These quantitative findings align with qualitative results from a conducted among BC childcare providers who emphasized the importance of staff training, role modelling and peer support as facilitators for implementing the AP Standards [32].

When examining predictors of practice changes, some factors within the inner setting (5 out of 9 factors) as well as characteristics of the providers (4 out of 8 characteristics) were associated with changes in PA/sedentary practices. For example, organizational climate, PA capacity related to materials, greater implementation support, higher self-efficacy related to PA as well as previous Appetite to Play training were each associated with positive practice changes related to FMS programming. The Appetite to Play workshop and materials focus on playful activities aimed at developing FMS and specifically targets providing activities in small spaces or with little to no equipment. Our findings align with a quantitative evaluation of the Appetite to Play initiative in which care providers reported increased knowledge and confidence related to physical literacy and PA [18]. Collectively, these results demonstrate the importance of supporting care providers with activities and ideas to support FMS development in a variety of indoor and outdoor spaces.

There were some unexpected findings. We found that greater capacity for space and equipment was associated with a decrease in practices related to the amount AP and outdoor AP. While these findings were surprising, this could be related to the fact that those centers that lacked adequate space and equipment were those centers who benefited the most from the Appetite to Play initiative, which had a specific focus on helping providers in incorporating more active play opportunities despite limits on space and equipment [18].

This study had several strengths including a longitudinal design which took advantage of a natural policy experiment in BC. Nonetheless, several limitations deserve consideration. First, all policies and practices were self-reported and could therefore be subject to social desirability bias. The items used to assess written policies and practices of services and the self report nature of assessment could have introduced bias. Observational data and a thorough document review of written policies would have provided additional estimates of implementation but would have made it difficult to obtain a province-wide cohort of centers across BC. However, we included both manager and staff responses which may present a more reliable depiction of policy and practices. Second, many factors were removed from the regression models due to multicollinearity issues. This may reflect measurement issues that need further consideration before assessing their potential influence in future implementation studies [31]. Finally, this study was limited to group childcare centers and the relatively small sample of centers makes it difficult to generalize to all childcare providers in BC.

## Conclusions

Limited Canadian studies have examined obesity prevention policy implementation within the childcare context, but available evidence suggests that fewer than half of Canadian childcare centers have written PA policies with considerable variations across provinces [12, 13]. Limited research also suggests that while many centers have policies in place, many lack resources and processes to support and enable policy implementation [33]. Therefore, interventions are needed to develop and effectively implement obesity prevention efforts within childcare settings.

Consistent with the socioecological model and Damschroder’s CFIR implementation science framework [19], we found that factors at the provider and organisational levels were associated with changes in PA policy and practices in a cohort of childcare centers in BC. Together with other studies [34, 35], our findings highlight the need to target factors at the organizational- (PA capacity related to staffing, commitment from staff to the AP Standards and level of institutionalization of policies) and provider- (self-efficacy toward PA) levels to improve childcare PA environments in BC. Future intervention research undertaken in real-world contexts is needed to test and optimize strategies to improve implementation of evidence-base PA interventions.

### Supplementary Information

**Additional file 1. **

**Additional file 2. **

## Data Availability

The datasets used and/or analysed during the current study available from the corresponding author on reasonable request.

## Abbreviations

AP: Active Play
BC: British Columbia
CFIR: Consolidated Framework for Implementation Research
EPAO-SR: Environment and Policy Evaluation and Observation Self-Report
PA: Physical activity
FMS: Fundamental movement skills
OR: Odds ratio
SD: Standard deviation
