# Supplementary material for: Factors associated with physical activity policy and practice implementation in British Columbia’s childcare settings: a longitudinal study
Source: BMC Public Health. 2023 Aug 29;23:1651. doi: 10.1186/s12889-023-16502-0 (PMC10463809; doi:10.1186/s12889-023-16502-0)
Supplement: Supplementary file 1 — Additional file 1. [file 12889_2023_16502_MOESM1_ESM.docx]

**Additional File 1**. Results from Confirmatory Factor Analyses*

| **Constructs** | **Items** | Factor Loadings | |
| --- | --- | --- | --- |
| **2-factor structure: Organizational Climate and Physical Activity (PA) Culture:** (Χ^2^(df=19)=60.17, p=.000; RMSEA=.066 (95%CI .05 - .09, p=.070; CFI.967; SRMR=.042) | |  | |
| Organizational climate  (α=.72) | Collegial … hostile | .64 | |
|  | Accepting of Change … Cautious to change | .69 | |
|  | Unsupportive … Supportive | .42 | |
|  | Friendly … Unfriendly | .86 | |
| PA culture  (α=.82) | The Active Play standards closely aligns with the core values and philosophy of our childcare facility | .81 | |
|  | In our childcare, staff prioritizes fundamental movement skill activities daily | .67 | |
|  | We strive to provide more physical activity than is recommended by the Active Play standards | .63 | |
|  | The Active Play standards and Appetite to Play resources fit well with our facility’s goals and objectives regarding physical activity and promotion of fundamental skills | .83 | |
| R between organizational climate and PA culture = .13 | | | |
| **3-factor structure: Physical Activity Capacity CFA:** (Χ^2^(df=10)=32.80, p=.000; RMSEA=0.074 (95%CI .05 - .10), p=.069; CFI=.969, SRMR=.-038) | |  | |
| PA capacity (space & equipment)  (α=.67) | We have all the necessary space and equipment to provide physical activity according to the Active Play standards | .73 | |
|  | We have all the necessary indoor space to provide physical activity  according to the Active Play Standards. | .61 | |
|  | We have all the necessary outdoor space to provide physical activity according to the Active Play Standards. | .73 | |
| PA Capacity (time)  (α=.70) | Our schedule easily allows staff to provide 120 min of physical activity during the day. | .78 | |
|  | Our schedule easily allows us to incorporate 60 min of outdoor play. | .66 | |
| PA Capacity (time)  (α=.68) | We have a staff who can provide engaging fundamental movement skill activities. | .87 | |
|  | We have someone who champions physical activity initiatives. | .63 | |
| r between PA capacity, space & equipment with time = -.53  r between PA capacity, space & equipment with staff = -.54  r between PA capacity, time with staff = .52  r between error terms indoor and outdoor time items for PA capacity (space & equipment) = .49. | | | |
| **2-factor structure: Implementation Support and Level of Institutionalization:** (Χ^2^(df=33)=87.04, p=.000; RMSEA=.07 (95%CI .05 - .09), p=.041; CFI=.957; SRMR=.043) | |  | |
| Implementation support  (α=.84) | Our childcare rewards staff who present new ideas/suggestions to meet the Active Play Standards. | .62 | |
|  | Staff regularly discuss ways they can meet the Active Play standards. | .81 | |
|  | [I am / The manager is] actively involved in planning activities that support active play and fundamental skill development. | .54 | |
|  | [I have / The manager has] allocated resources to train staff to learn ways to engage children in fundamental movement skill activities. | .68 | |
|  | [I / The Manager] regularly monitors and provides feedback to staff on how to better meet Active Play standards. | .78 | |
|  | To meet the Active Play Standards, our facility recently held a meeting to discuss it. | .64 | |
|  | Our facility has purchased equipment and/or re-organized our space to support the implementation of the Active play Standards. | .54 | |
| Level of institutionalization  (α=.75) | In the past year, our facility has changed physical activity policies/guidelines related to the time children spend outdoors, and fundamental movement skill development. | .46 | |
|  | In the past year, our facility has changed policies/guidelines related to the time children spend sitting. | .50 | |
|  | In the past year, our facility has implemented a policy that ensured all staff were given regular professional development to support the Active Play Standards. | .93 | |
| r between Implementation support with Level of institutionalization = .51  r between error terms for items assessing whether the facility has changed their policy regarding time children spend sitting and whether the facility has a policy related to staff receiving professional development to support implementation of the Active Play Standards = .58) | | | |
| **4-factor structure: Attributes of the Innovation**: (Χ^2^(df=29)=129.06, p=.000; RMSEA=.083 (95%CI .06 - .10), p=.000; CFI=.960; SRMR=.042) | | | |
| Flexibility/triability (α=.77) | The AP Standards are flexible to implement. | | .69 |
|  | There are many ways I can implement and meet the AP Standards. | | .82 |
|  | It was easy to test out ways of meeting the AP Standards. | | .69 |
| Outcome expectations (α=.80) | Implementation of the AP Standards and activities can improve children FMS. | | .91 |
|  | Implementation of the AP Standards and activities can increase children’s interest. | | .92 |
|  | Children in our childcare respond well to and enjoy AP time. | | .52 |
| Relative advantage (α=.79) | I believe that the new Active Play Standards plus Appetite to Play resources are more effective in improving physical activity level and fundamental movement skills than past practice. | | .92 |
|  | I believe that the Active Play Standards plus Appetite to Play resources are easier to use than other physical activity or skill programs. | | .76 |
| Acceptability (α=.79) | I find the ATP resources appealing. | | .82 |
|  | The ATP resources and materials are implementable into daily practice. | | .80 |
| **1-factor structure: Self-efficacy:** (Χ^2^(df=2)=24.63, p=.000; RMSEA=.181 (95%CI .12 - .25), p=.000; CFI=.939; SRMR=.051) | | | |
| Self-efficacy (α=.77) | I am capable of planning daily FMS activities according to the AP Standards. | | .59 |
|  | At all times, I am capable of providing children with 60 min of outdoor AP time. | | .77 |
|  | Regardless of the schedule, I am capable of providing a total of 120 min per day of combined indoor or outdoor active play to children in my facility. | | .74 |
|  | At all times, I am capable of limiting children’s sitting to less than 60 min at a time. | | .61 |

* Overall fit of the CFA were analyzed using multiple criteria as there are no agreed standards. The following criteria were evaluated and used to determined if the solution was deemed a good fit. RMSEA (Steiger’s Root Meant Square Error of Approximation with and upper 95% CI less than .08 or .10; CFI (Comparative Fit Index) >=.95; SRMR (Standardized Root Mean Square Residual) <=.05.

r = correlation
