# Supplementary material for: Factors associated with physical activity policy and practice implementation in British Columbia’s childcare settings: a longitudinal study
Source: BMC Public Health. 2023 Aug 29;23:1651. doi: 10.1186/s12889-023-16502-0 (PMC10463809; doi:10.1186/s12889-023-16502-0)
Supplement: Supplementary file 2 — Additional file 2. [file 12889_2023_16502_MOESM2_ESM.doc]

STROBE Statement—Checklist of items that should be included in reports of ***cohort studies***

|  | Item No | Recommendation |
| --- | --- | --- |
| **Title and abstract** | 1 | (*a*) Indicate the study’s design with a commonly used term in the title or the abstract  *Yes* |
|  |  | (*b*) Provide in the abstract an informative and balanced summary of what was done and what was found  *See p. 2-3* |
| Introduction | | |
| Background/rationale | 2 | Explain the scientific background and rationale for the investigation being reported  *See p. 4-7* |
| Objectives | 3 | State specific objectives, including any prespecified hypotheses  *See p. 7. The aim of this paper was to explore factors associated with implementation of four types of PA and sedentary policies and practices specifically targeted by these Standards: FMS, amount of total AP, limits on screen time, and amount of outdoor AP.* |
| Methods | | |
| Study design | 4 | Present key elements of study design early in the paper  *See p. 7: “This study used data from a longitudinal cohort embedded within a larger repeat cross-sectional study, which surveyed managers and staff from licensed group childcare facilities before and after the enforcement of the AP Standards.”* |
| Setting | 5 | Describe the setting, locations, and relevant dates, including periods of recruitment, exposure, follow-up, and data collection  *See p. 7-8.* |
| Participants | 6 | (*a*) Give the eligibility criteria, and the sources and methods of selection of participants. Describe methods of follow-up  *See line 183-186: “Respondents were eligible to participate if their facility cared for children aged 2 ½ -5 years old, was licensed for group childcare, preschool (offering full days), and/or multi-age childcare, and if the respondent was a manager or a staff overseeing or caring for children aged 2 ½ - 5 years old.”* |
|  |  | (*b*) For matched studies, give matching criteria and number of exposed and unexposed  *Not applicable.* |
| Variables | 7 | Clearly define all outcomes, exposures, predictors, potential confounders, and effect modifiers. Give diagnostic criteria, if applicable.  *See lines 192-313.* |
| Data sources/ measurement | 8* | For each variable of interest, give sources of data and details of methods of assessment (measurement). Describe comparability of assessment methods if there is more than one group.  *See lines 192-313. See also Table 1 & Supplementary table 1.* |
| Bias | 9 | Describe any efforts to address potential sources of bias  *We also used a response scale when asking about the presence of policies to distinguish between written vs. non-written but more informal policies. We tried to limit response bias by sending the survey to multiple participants (managers and staff within a facility). When more than one manager or more than one staff from the same facility responded to the survey, the policy and practice scores were averaged across all respondents by role (i.e., across all managers or across all staff).*  *We also explicitly address the potential for social desirability bias in the Discussion (see limitation section lines 412-480)* |
| Study size | 10 | Explain how the study size was arrived at  *See lines 357-360.* |
| Quantitative variables | 11 | Explain how quantitative variables were handled in the analyses. If applicable, describe which groupings were chosen and why  *See lines 192-313.* |
| Statistical methods | 12 | (*a*) Describe all statistical methods, including those used to control for confounding  *See lines 320-355.* |
|  |  | (*b*) Describe any methods used to examine subgroups and interactions  *Not applicable.* |
|  |  | (*c*) Explain how missing data were addressed  *See lines 349-352.* |
|  |  | (*d*) If applicable, explain how loss to follow-up was addressed  *Not applicable* |
|  |  | (*e*) Describe any sensitivity analyses  *Not applicable.* |
| Results | | |
| Participants | 13* | (a) Report numbers of individuals at each stage of study—eg numbers potentially eligible, examined for eligibility, confirmed eligible, included in the study, completing follow-up, and analysed  *See lines 357-360.* |
|  |  | (b) Give reasons for non-participation at each stage  *Not applicable.* |
|  |  | (c) Consider use of a flow diagram  *Not applicable.* |
| Descriptive data | 14* | (a) Give characteristics of study participants (eg demographic, clinical, social) and information on exposures and potential confounders  *See Table 2* |
|  |  | (b) Indicate number of participants with missing data for each variable of interest  *See Table 2* |
|  |  | (c) Summarise follow-up time (eg, average and total amount)  *See Table 2* |
| Outcome data | 15* | Report numbers of outcome events or summary measures over time  *See Table 2* |
| Main results | 16 | (*a*) Give unadjusted estimates and, if applicable, confounder-adjusted estimates and their precision (eg, 95% confidence interval). Make clear which confounders were adjusted for and why they were included  *See Table 2 & table 3-4 for adjusted estimates.* |
|  |  | (*b*) Report category boundaries when continuous variables were categorized  *See Table 2* |
|  |  | (*c*) If relevant, consider translating estimates of relative risk into absolute risk for a meaningful time period  *Not applicable.* |
| Other analyses | 17 | Report other analyses done—eg analyses of subgroups and interactions, and sensitivity analyses  *Not applicable.* |
| Discussion | | |
| Key results | 18 | Summarise key results with reference to study objectives  *See lines 410-420.* |
| Limitations | 19 | Discuss limitations of the study, taking into account sources of potential bias or imprecision. Discuss both direction and magnitude of any potential bias  *See limitations lines 471-483* |
| Interpretation | 20 | Give a cautious overall interpretation of results considering objectives, limitations, multiplicity of analyses, results from similar studies, and other relevant evidence  *See lines 410-470*. |
| Generalisability | 21 | Discuss the generalisability (external validity) of the study results  *See lines 481-483.* |
| Other information | | |
| Funding | 22 | Give the source of funding and the role of the funders for the present study and, if applicable, for the original study on which the present article is based  *See lines 526-531.* |

*Give information separately for exposed and unexposed groups.

**Note:** An Explanation and Elaboration article discusses each checklist item and gives methodological background and published examples of transparent reporting. The STROBE checklist is best used in conjunction with this article (freely available on the Web sites of PLoS Medicine at http://www.plosmedicine.org/, Annals of Internal Medicine at http://www.annals.org/, and Epidemiology at http://www.epidem.com/). Information on the STROBE Initiative is available at http://www.strobe-statement.org.
